# 

**Published:** 1874-08

**Authors:** 


### ASSOCIATE EDITORS:

**GEORGIA.**

James A. Long, M.D.

W. L. M. Harris, M.D.

H. L Battle, M.D.

W. C. Musgrove. M D

L B. Bouchelle, M.D.

Thomas Burdell, M.D.

E. H. W. Hunter, M.D.

V. S Hitt, M.D.

J. E. Pope, M.D.

A. H. Brantley. M.D.

J. J. Knott, M.D.

J. C. Wisdom, M.D.

W. W. Leake, M.D.

S. G. WHITE, M.D.

W. H. [illegible], M.D.

G. D CASE, M.D.

**KENTUCKY.**

W. S. Taylor, M.D.
B. W. Stone, M.D.
Charles Kearns, M.D.

**TEXAS.**

S. M. Welch, M.D.
T. J. Heard, M.D.
W. A. Heard, M.D.

**TENNESSEE.**

J. D. Tucker, M.D.

**ALABAMA.**

J. C. Knox, M.D.
Geo. F. Taylor, M.D.
J. B. Barnett, M.D.
S. M. Hogan, M.D.
D. S. Hopping, M.D.
J. T. Searcy, M.D.
S. F. Hill, M.D.

**NORTH CAROLINA.**

J. B. Hughes, M.D.
S. S. Satchwell, M.D.
A. B. Pierce, M.D.
H. Kelly, M D.
Geo. A. Foote, M.D.
E. A. Anderson, M.D.
H. T. Bahnson, M.D.
Willis Alston, M.D.
Thos. F. Wood, M.D.
N. J. Pittman, M.D.
John W. [illegible], M.D.

**SOUTH CAROLINA.**

E. T. McSwain, M.D.
G. M. Rivers, M.D.

**OHIO.**

J. Q. A. Hudson, M.D.
C. H. Tidd, M.D.

**VIRGINIA.**

John H. Claiborne, M.D.
F. Horner, Jr., M.D.
R. J. Preston, M.D.
J. E. Lockridge, M.D.
J. S. Wharton, M.D.
Wm. O. Owen, M.D.
Sam'l P. Ripley, M.D.
Benj. Blackford, M.D.
E. A. Drewry, M.D.
Rob B. Tunstall, M.D.
W. F. Barr, M.D.
L. B. Anderson, M.D.
J. M. Lazzell, M.D.

**MISSISSIPPI.**

C. B. Gallaway, M.D.
Edward Lea, M.D.
S. V. D. Hill, M.D.
E. T. Henry, M.D.
T. P. Lockwood, M.D.
Robert Kells, M.D.

**ARKANSAS.**

A. D. Hodge, M.D.

J. K. Hodge, M.D.

A. W. Hobson, M.D.

**NEW JERSEY.**

J. B. MATTISON, M.D.

### CORRESPONDING EDITORS:

Ely Van DeWarker, M. D., New York.
C. C. F. Gay, M.D., New York, *Surgeon of Buffalo General Hospital.*
M. H. Henry. M.D., New York, *Surgeon to N. Y. Dispensary, Dep't of Venerial and Skin Diseases.*
Benjamin Howard, M.D., New York.
James Tyson, M.D., Philadelphia, Pa.
W. W. Keen, M.D. Philadelphia.
Robert Robson, M D., Indiana.
A. Patton, M.D., Indiana.
John H. Weir, M.D., Illinois.
Thomas J Gallaher, M. D, Pennsylvania.
S. C. Busey, M.D., Washington, D. C.
Wm. R. Whitehead, M.D., Colorado Territory.

